# Numerical Simulation Data-Aided Domain-Adaptive Generalization Method for Fault Diagnosis

**DOI:** 10.3390/s25113482

**Published:** 2025-05-31

**Authors:** Tao Yan, Jianchun Guo, Yuan Zhou, Lixia Zhu, Bo Fang, Jiawei Xiang

**Affiliations:** 1 College of Mechanical and Electrical Engineering, Wenzhou University, Wenzhou 325035, China20202019@wzu.edu.cn (L.Z.); 2 Ningbo Puer Mechanical Electrical Manufacturing Co., Ltd., Yuyao 315420, China; yyihc@163.com; 3 Wenzhou Key Laboratory of Advanced Equipment Dynamics and Intelligent Diagnosis-Maintenance, Wenzhou 325035, China

**Keywords:** finite element model, domain generalization, fault diagnosis, domain adaptive

## Abstract

In order to deal with the cross-domain distribution offset problem in mechanical fault diagnosis under different operating conditions. Domain-adaptive (DA) methods, such as domain adversarial neural networks (DANNs), maximum mean discrepancy (MMD), and correlation alignment (CORAL), have been advanced in recent years, producing notable outcomes. However, these techniques rely on the accessibility of target data, restricting their use in real-time fault diagnosis applications. To address this issue, effectively extracting fault features in the source domain and generalizing them to unseen target tasks becomes a viable strategy in machinery fault detection. A fault diagnosis domain generalization method using numerical simulation data is proposed. Firstly, the finite element model (FEM) is used to generate simulation data under certain working conditions as an auxiliary domain. Secondly, this auxiliary domain is integrated with measurement data obtained under different operating conditions to form a multi-source domain. Finally, adversarial training is conducted on the multi-source domain to learn domain-invariant features, thereby enhancing the model’s generalization capability for out-of-distribution data. Experimental results on bearings and gears show that the generalization performance of the proposed method is better than that of the existing baseline methods, with the average accuracy improved by 2.83% and 8.9%, respectively.

## 1. Introduction

As essential components in rotating machinery, bearings are extensively utilized across various industries such as manufacturing, aerospace, automotive, energy, and heavy industry. Due to their frequent operation in challenging conditions, they often fail before reaching their anticipated service life. This not only results in equipment downtime and production losses but can also cause serious injuries and significant economic losses. Therefore, monitoring the health of rotating machinery components is crucial. This can both ensure the normal operation of the machinery and serve as an important measure to protect the safety of the operator [1,2,3,4].

Over the past few years, deep learning (DL) technology has achieved notable advancements in the rotating machinery fault diagnosis area, and the wide range of applications have demonstrated great potential. The raw data are processed end-to-end by a DL model and directly converted into fault diagnosis results. This end-to-end processing method greatly simplifies the traditional diagnostic process by eliminating the need for extensive feature engineering and expert intervention. Owing to its efficiency and high degree of automation, DL is rapidly gaining recognition and substantial traction in rotating machinery fault diagnosis. Li et al. [5] designed an end-to-end adaptive multi-scale fully convolutional network (AMFCN) for bearing fault diagnosis in various signal-to-noise ration (SNR) environments. Wen et al. [6] utilized a convolutional neural network (CNN) with a hierarchical structure to evaluate the fault location and fault severity of mechanical devices. Xu et al. [7] developed a hybrid DL model based on a CNN and deep forest (gcForest). Wang et al. [8] introduced a batch normalization method at each layer of the deep neural network (DNN). Although DL-based methods have made significant strides in intelligent fault diagnosis, the literature [9] points out that they need to assume that the distribution of training and test data is consistent. This assumption poses a serious limitation in engineering practice. Obtaining sufficient and balanced data remains a key challenge for intelligent diagnosis. Notably, the progress of adaptive variational autoencoding generative adversarial networks (AVAEGANs) [10] and traceable multi-domain collaborative generative adversarial networks (TMCGANs) [11] has made substantial progress in addressing this issue. Due to the complexity and variability of operating environments, rotating components frequently operate under diverse conditions. This variability leads to differences in data distribution, thereby weakening the diagnostic performance of the models [12]. Therefore, devising robust diagnostic techniques to meet the challenges of domain shift is vital for enhancing the reliability of rotating machinery.

Transfer learning (TL), an emerging artificial intelligence (AI) method, leverages knowledge from one domain to facilitate learning in other related but distinct domains. This method eliminates the need for training and test data to align with the same distribution, thereby significantly reducing application limitations. Among them, DA-based methods are particularly widely used [13]. Aiming at this aspect, an increasing number of scholars have employed TL methods for intelligent fault diagnosis, achieving notable results. Guo et al. [14] designed a deep convolutional transfer learning network (DCTLN) to solve the fault diagnosis problem under diverse working conditions. Zhang et al. [15] presented a Wasserstein distance to guide a multi-adversarial network (WDMAN) for promoting effective representation learning of source and target domains. In [16], a novel joint distribution adaptation mechanism (IJDA) was introduced, which combined MMD and correlation alignment (CORAL) as a new distribution difference index.

DA-based methods can effectively utilize some target domain data during the training stage, thereby demonstrating excellent performance in the target domain. However, rotating machinery often works in a constantly changing operating environment, making the acquisition of target data in advance very difficult. In this case, DA methods cannot be used due to the lack of available target domain data. Therefore, it is of great interest and necessity to explore a generalized diagnostic method that can be trained only on the source domain data without accessing any target data, but can still effectively solve the problem of unseen target data. This aspect is equally critical for diagnostic tasks performed in real time. The aforementioned method is referred to as domain generalization (DG) [17]. DG has also attracted growing interest in intelligent diagnosis. Two commonly employed techniques in DG are enhancing data diversity and learning representations that are invariant across domains. The former aims to enhance the diversity and usability of training data through the augmentation and extension of the input data. The most commonly used is the generative adversarial network (GAN) [18]. In addition, numerical simulation methods have become a common tool in intelligent diagnostic research because they can efficiently simulate complex system behaviors and fault characteristics. Zheng et al. [19] developed a numerical simulation-enhanced RV reducer fault diagnosis method. Wang et al. [20] combined numerical simulation models with machine learning to implement the online fault diagnosis of bearings. Xu et al. [21] solved the problem of insufficient fault sample data by establishing a connecting rod-fastening rotor dynamics model. The above numerical simulation method effectively alleviates the challenges brought about by data scarcity in intelligent diagnostic research and provides important data support for model development and performance verification. However, there is little research on applying simulation data augmentation to DG. For example, the literature review [22] of DG pointed out in its future work that using simulation models to generate simulation data has great potential for application in DG. The latter aims to learn feature representations that are stable and consistent across domains, and centers on training models to extract features that are valid across different data distributions. For example, the research conducted by [23] introduced an intelligent fault detection approach utilizing multiple source domains, with the goal of effectively extracting domain-invariant characteristics from diverse source domains. Shi et al. [24] presented an intelligent fault diagnosis method for unknown conditions built upon a domain generalization theory. In contrast, Chen et al. [25] developed a general adversarial domain invariant generalization (ADIG) regression framework to address the issue of feature migration between different domains. Hu et al. [26] designed a novel loss for domain generalization. In addition, both federated learning and meta-learning have shown promising results in improving domain generalization capabilities. For example, recent studies [27,28,29,30] explored the potential of meta-learning and federated learning in DG, which makes both methods particularly useful in the case of small samples. Among them, Jian et al. [29] designed a domain generation module to generate data with different distributions and used meta-learning to simulate domain shift. These methods have demonstrated their effectiveness in handling previously unseen target data, thus paving the way for a new research trajectory within the realm of intelligent diagnosis. However, based on the above methods, the model still needs sufficient source domain training data to ensure good performance in the target domain.

Overall, DG can effectively tackle fault diagnosis in scenarios where the target domain is unseen. However, how to obtain a substantial quantity of source domain data and effectively learn domain-invariant feature representations to support DG based on multi-source domains is still a problem to be solved. To overcome this difficulty, this study applies simulation data to DG for the first time. It constructs multi-source domains by combining simulation data with measured data, facilitating the model to learn more domain-independent features through adversarial training, thereby generalizing effectively to unseen target domains. The key contributions of this study are outlined below:

1. We propose a DG method augmented by numerical simulation data, where simulation data representing different operating conditions are used together with measured data from other different conditions. Increasing domain diversity by introducing different speeds and loads improves generalization performance across unknown domains.
2. The proposed method is superior to the traditional DG method without simulated data augmentation. Experimental verification shows that with the augmentation of simulated data, the generalization performance of the adversarial training model on the unknown target domain is effectively improved.

The remaining sections of this article are organized as follows: Section 2 details the basic theory of transfer learning. Section 3 details the general framework of the proposed method. Section 4 is the experimental design and result analysis. Section 5 is the summary of the paper.

## 2. Basic Theory

### 2.1. Domain Adaptation

DA is a popular direction in TL research. It mainly focuses on the case where the source and target domains have the same feature space and label space $\left(X_{s}=X_{t},Y_{s}=Y_{t}\right)$, but the probability distributions of the data are different $\left(P_{s}\left(x,y\right)\neq P_{t}\left(x,y\right)\right)$. The fundamental concept of DA involves leveraging the knowledge acquired in the source domain. This enables the model to perform effectively in the target domain through adaptation and adjustment. DA is widely used in fault diagnosis, and methods such as [31,32,33,34] have made remarkable progress.

The traditional DA method first measures the similarity of feature distribution between the source domain and target domain and quantitatively calculates the similarity degree between them. Then, using this metric as an optimization criterion, the features of the two domains are mapped to a common feature space by global local transformation to improve their similarity. Through this process, features shared between the two domains can be extracted. One of the most frequently utilized distance metrics in DA issues is MMD. MMD evaluates the similarity of two domains by measuring the difference between their mean embedding distributions, defined as follows:

(1) $$MMD_{H}\left(p,q\right)={\left\|E_{q}\left[\varphi \left(x^{s}\right)\right]-E_{p}\left[\varphi \left(x^{t}\right)\right]\right\|}_{H}^{2}$$

where $x^{s}$ and $x^{t}$ denote the source domain sample features and target domain sample features, respectively; q, p refers to the feature distributions of the source domain $x^{s}$ and the target domain $x^{t}$, respectively; H stands for a reproducing kernel Hilbert space; $\varphi \left(\cdot \right)$ signifies the feature mapping function to which the original sample features are mapped; and $E\left[\cdot \right]$ indicates the mathematical expectation calculation function.

### 2.2. Domain Generalization

Training data S are from *N* domains with different but similar data distributions: $S={\left\{S^{i}\right\}}_{i=1}^{N}$. We assume that each domain contains *M* samples where each domain $S_{i}={\left\{\left(x_{j},y_{j}\right)\right\}}_{j=1}^{M}$, $\left(x_{j},y_{j}\right)\sim P_{i}\left(x,y\right)$ indicates that each domain datum obeys its own data distribution $P_{i}$. The objective of DG is to train a model f: x→ℝ from these *N* domains such that the prediction error of f on the test data constructed from these *N* domains $S_{t}={\left\{\left(x_{i}\right)\right\}}_{i=1}^{N_{t}}$ is minimized, i.e., making $\frac{1}{N_{t}}\sum_{i=1}^{N_{t}}l\left(f\left(x_{i}\right),y_{i}\right)$ obtain the minimum value. It should be noted that since the DG problem does not have a target domain like the DA problem, its test data $S_{t}$ are constructed by sampling from the existing *N* training data. However, the problem of DG is more challenging in practice because of the inaccessibility of the target data. One of the most straightforward base models should be training on a mixture of data from all domains, which in turn learns a model f that has been trained on all data. This can be accomplished by optimizing the objective function that follows:

(2) $$f^{⋆}=\underset{f}{\mathrm{argmin}}\frac{1}{MN}\overset{MN}{\underset{j=1}{\sum }}l\left(f\left(x_{j}\right),y_{j}\right)$$

where $l\left(\cdot ,\cdot \right)$ denotes a specific loss function, such as cross entropy loss.

**Figure 1:**
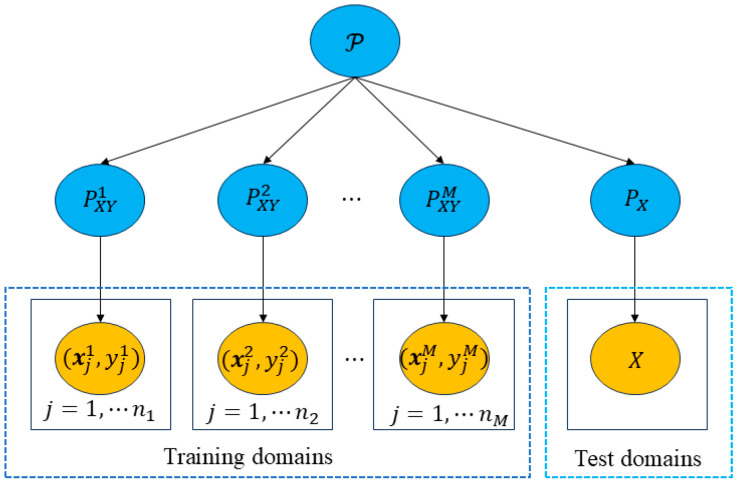
Sourced from [35]. Overview of DG.

Wang et al. [35] detailed existing DG methods and categorized them into three types: data manipulation, representation learning, and learning strategies. Data manipulation mainly increases the diversity and availability of training data through techniques such as data augmentation and data generation; representation learning aims to keep the model consistent under different data distributions by learning domain invariant feature representations. Learning strategies are crafted to boost the generalization performance of the model through the implementation of specialized learning techniques. The basic elements of DG are shown in Figure 1.

### 2.3. Adversarial Training

Adversarial training can effectively improve the model’s robustness to input perturbations and its generalization ability on out-of-distribution data by introducing adversarial examples and adversarial loss, thereby significantly improving the model’s performance in complex or unknown environments. It is a very important training method in DL at present. Through a continuous game between the generative network (*G*) and the discriminative network (*D*), the generative adversarial network (GAN) enables the *G* to gradually approach and perfectly match the distribution of real data. Throughout the training process, *G* strives to create highly realistic data to fool the *D*. Conversely, the *D* endeavors to accurately differentiate between the genuine data and the false data produced by the G, which plays the following minimax game between two people:

(3) $$\min_{G}\max_{D}V\left(D,G\right)=E_{x\sim P_{data}}\log D\left(x\right)+E_{z\sim P_{z}\left(z\right)}\log(1-D\left(G\left(z\right)\right)$$

where x and z refer to the actual instances and random noise, respectively. When $P_{g}=P_{data}$, the minimax game achieves its global optimum [24].

Adversarial training has become a widely adopted technique for addressing various data processing challenges. By introducing a competitive framework between two models, adversarial training enhances the robustness and performance of machine learning algorithms. In [36], a domain adversarial neural network (DANN) composed of feature extractor *G*, label predictor *C*, and domain discriminator *D* is proposed. The DANN learns domain-invariant representations by engaging in a competitive game between the *G* and the *D*, and it has the training objectives outlined below:

(4) $$\min_{G,C}\max_{D}V(G,C,D)=\underset{x\in D_{S}}{\sum }L_{y}(G,C)-\lambda \underset{x\in D_{S}\cup D_{T}}{\sum }L_{d}\left(G,D\right)$$

where $L_{y}$ is the loss function of the label predictor C, and $L_{d}$ is the loss function of the domain classifiers D.

Over the past few years, adversarial learning has seen a surge in popularity within the domain of cross-domain intelligent fault diagnosis. Its strong adaptive capability and potential to learn domain-invariant features under different data distributions make it excel in solving complex diagnostic tasks. Through the interaction of the source domain and the target domain, adversarial learning effectively extracts robust feature representations, thereby increasing the diagnostic performance of the model across varying operating conditions [37,38,39].

## 3. The Proposed Method

**Figure 2:**
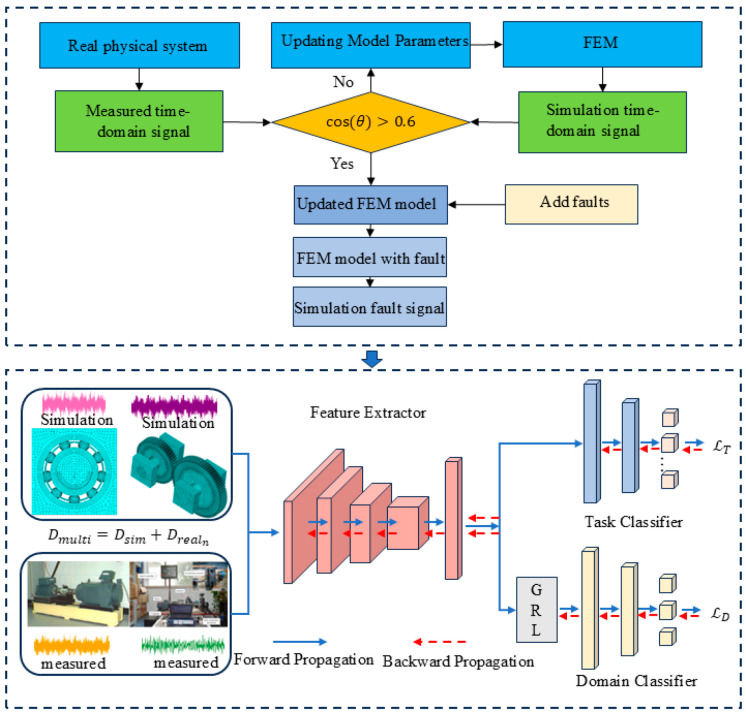
Framework of the proposed method.

This paper mainly solves the problem of source domain data scarcity in multi-source domain generalization by constructing an FEM to generate simulation data as an enhanced domain and combining it with the real domain. Figure 2 shows the overall flow chart of the article. Table 1 shows the main parameters of the network.

### 3.1. Finite Element Model

Building on the existing research in the laboratory [40] and incorporating the concept of DG, an innovative DG method utilizing simulation data enhancement is introduced to effectively address the issue of limited source domains in multi-source domain adversarial learning processes. The general steps of this paper are as follows:

Step 1: Establish an FEM of the initial healthy state, simulate the dynamic response under such a state, and obtain the simulation signal of the healthy state.

Step 2: To make the vibration response signal generated by the FEM highly match the system vibration response signal of the actual physical model under a running state, the cosine similarity is used as a matching metric tool to modify the FEM. The closer the cosine similarity is to 1, the more effective the model is. Generally, satisfactory results will be achieved when cosine similarity is greater than 0.6 [41].

(5) $$\cos\theta =\frac{V_{p}V_{s}}{\left\|V_{p}\|\|V_{s}\right\|}=\frac{\sum_{i=1}^{N}V_{p}^{i}V_{s}^{i}}{\sqrt{\sum_{i=1}^{N}{\left(V_{p}^{i}\right)}^{2}}\sqrt{\sum_{i=1}^{N}{\left(V_{s}^{i}\right)}^{2}}}$$

where *V_p_* and *V_s_* are the vibration responses of length *N* obtained by the physical system test and numerical model simulation, respectively.

Step 3: Based on the modified FEM, faults are added to construct an FEM with fault characteristics to obtain simulation data.

Through the above steps, an FEM from a healthy state to a fault state is constructed to ensure that the simulation signal is highly consistent with the real signal in the healthy state and successfully generates high-fidelity simulation signals of various typical faults, preparing for the subsequent construction of simulation-enhanced multi-source domains.

### 3.2. Multi-Source Domain Construction

In the finite element modeling process, we ensured the accuracy of the simulation model through strict modeling and correction steps. At the same time, cosine similarity was used as an evaluation index to verify that there was a high similarity between the simulation data and the measured data. The generation of fault data is based on a corrected high-precision model and has good credibility. Therefore, it can be considered that the current simulation data have high reliability and can reduce the risk of the model being biased towards simulation data.

The simulation working condition data $D_{sim}$ ($D_{sim}=\{\left(x_{sim}^{i},y_{sim}^{i}\right)|i=1,2,\ldots ,N_{sim}\}$) are obtained through the FEM. Among them, $x_{sim}^{i}$ represents the simulation data sample, $y_{sim}^{i}$ represents the corresponding label, and $N_{sim}$ denotes the number of simulation data samples. $D_{real_{i}}$ represents the measured data of diverse working conditions. Constructing multi-source domain data $D_{multi}$, we obtain the follows:

(6) $$D_{multi}=D_{sim}\cup D_{real_{i}}\left(i=1,2\ldots N\right)$$

where *N* is the number of real domains. In order to distinguish data from diverse working conditions, the domain label *d* is introduced, and the multi-source domain dataset may be expanded to the following:

(7) $$D_{multi}=\{\left(x_{k},y_{k},d_{k}\right)|k=1,2,\ldots ,N_{sim}+N_{real_{i}}\}$$

where *k* and *i* represent the number of multi-source domains and the number of real domains, respectively.

Use the constructed multi-source domain data for domain adversarial training to achieve domain generalization. The model architecture consists of three parts: feature extractor *F*, task classifier *T*, and domain discriminator *D*. Randomly select samples from multi-source domain data and input them into the model. After passing through the feature extractor *F*, the extracted features *F*(*x*) are respectively transmitted to *T* and *D*. *T* maps *F*(*x*) to the corresponding category labels to learn the discriminative information. *D* performs domain confusion operation on *F*(*x*) to make it domain invariant. In domain adversarial training, task classification loss and domain discrimination loss are considered separately to make the learned features discriminative and domain invariant. For the original samples from $D_{multi}$, the task classification loss $L_{T}$ and the domain discrimination loss $L_{D}$ can be expressed as follows:

(8) $$L_{Task}=\frac{1}{n_{s}}\overset{K}{\underset{k=1}{\sum }}\overset{n_{k}}{\underset{i=1}{\sum }}l\left(T\left(F\left(x_{k}^{i}\right)\right),y_{k}^{i}\right)$$

(9) $$L_{Domain}=\frac{1}{n_{s}}\overset{K}{\underset{k=1}{\sum }}\overset{n_{k}}{\underset{i=1}{\sum }}l\left(D\left(R\left(F\left(x_{k}^{i}\right)\right)\right),d_{k}^{i}\right)$$

where $y_{k}^{i}$ and $d_{k}^{i}$ represent the category label and domain label, respectively. $l\left(\cdot \right)$ and $R\left(\cdot \right)$ are the cross-entropy loss and gradient reversal layer, respectively. $n_{s}$ denotes the number of source domain samples.

The total loss is expressed as follows:

(10) $$L_{Total}=L_{Task}-\lambda L_{Domain}$$

By alternately training the domain discriminator and the feature extractor, the model learns to distinguish between multi-source domains while simultaneously extracting features that are common across these domains. This process ultimately realizes the goal of domain adversarial training, enhancing the model’s robustness and reducing dependency on any specific domain.

## 4. Experimental Research

### 4.1. FEM Construction

#### 4.1.1. CWRU Bearing FEM

In this part, the simulated model is established based on the rolling bearing 6205 of CWRU. The three-dimensional FEM is established by using the dynamic module of finite element analysis software ANSYS (Version 17.0). During the finite element modeling process, SOLID164 solid cells were used to mesh the 3D body. In order to apply a rotating load, SHELL163 shell cells are used to mesh the inner surface of the inner ring. For calculation errors and calculation time, the SWEEP meshing method is employed to create a substantial number of finite cells. Considering the contact of bearings in the actual work, three contact pairs are established in the finite element model, namely the contact between the roller and inner ring, roller and outer ring, and cage and roller [40]. The cell size is 1 mm for the outer and inner rings and the rollers, 0.5 mm for the cage, and 2 mm for the shaft and housing. Through effective meshing, the FEM of the bearing contains 71,976 cells and 73,133 nodes. Table 2 provides the details of the established FEM parameters. The bearing faults were modeled by modifying the inner and outer race surfaces and rolling element geometry to simulate faults. Specifically, small pits with diameters of 0.007 inches, 0.014 inches, and 0.021 inches were introduced into the inner race, outer race, and ball surfaces to represent defects of varying degrees [40]. As for the types of bearing failures according to CWRU offerings, ten types of bearing faults were selected as the required faults and the corresponding vibration signals needed to be generated by the simulation. They include inner ring failure (IR), outer ring failure (OR), and rolling element failure (RE), respectively. Each failure contains three different degrees of damage (0.007, 0.014, and 0.021 inches). For example, IR007 represents a 0.007-inch fault on the inner race of a bearing.

**Figure 3:**
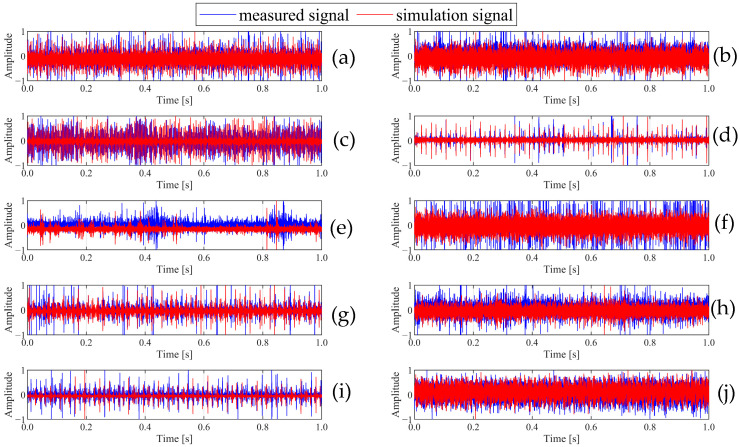
Time domain comparison of bearing fault simulation data and measured data: (**a**) normal, (**b**) IR007, (**c**) RE007, (**d**) OR007, (**e**) IR014, (**f**) RE014, (**g**) OR014, (**h**) IR021, (**i**) RE021, (**j**) OR021.

Figure 3a–j show the waveforms of ten faults in the time domain. Figure 3 clearly shows that the overall waveform shape of the simulation data is similar to that of the measured data, indicating that the simulated data can reflect the characteristics of the actual signal to a certain extent. The bearing fault diagnosis results in Section 4 effectively demonstrate the validity of the simulation data.

#### 4.1.2. Gearbox FEM

**Figure 4:**
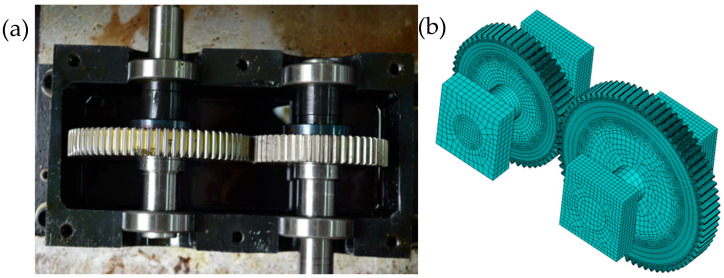
Single-stage gear train and finite element modeling. (**a**) Involute spur gears, (**b**) Finite element model of the gear transmission system.

Based on the laboratory single-stage gearing system, this study focuses on involute spur gears. The system includes a main gear, a driven gear, main and driven shafts, and four N205 cylindrical roller bearings, as shown in Figure 4a. We established a three-dimensional finite element model of a gear transmission system with finite element analysis software ANSYS. In the process of establishing the FEM of the gears, the SWEEP meshing method is also used to mesh the geometric model of the gears. Through effective mesh division, the finite element model of the gear transmission system was established, as presented in Figure 4b. According to the actual working conditions of the gears, five contact pairs were established in the FEM, including four contact pairs between the shaft and the support and one contact pair between the active gear teeth and the driven gear teeth, and all degrees of freedom of the four support surface nodes were restricted [42]. For the experimental gear train, the geometrical and material parameters of the gears are given in Table 3. During the experimental and simulation studies, the rotation speed was fixed at 1474 rpm and a reverse torque of 5 N/m acted as a drag load on the driven gears. The sampling frequency was set to 5120 Hz, and the acceleration signal was collected from the gearbox body at the drive end of the main shaft. By adding the desired faults to the normal model, the corresponding fault simulation signals were obtained.

**Figure 5:**
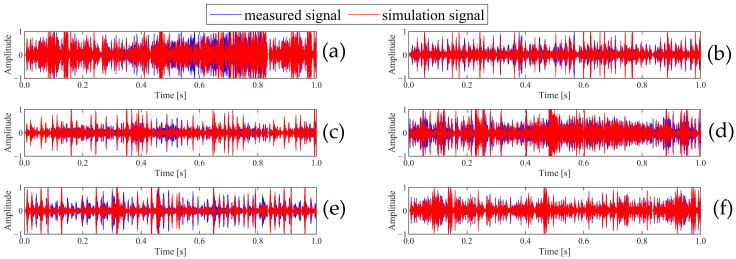
Time domain waveforms of gear fault simulation data and measured data. (**a**) DSP + DB, (**b**) DSP, (**c**) DB + DNB, (**d**) DB, (**e**) DC, (**f**) normal.

Figure 5a–f show the waveforms of the six gear states in the time domain. Figure 5 clearly shows that the overall trend of the simulation signal and the measured signal is basically consistent, but there are differences in some local details.

**Figure 6:**
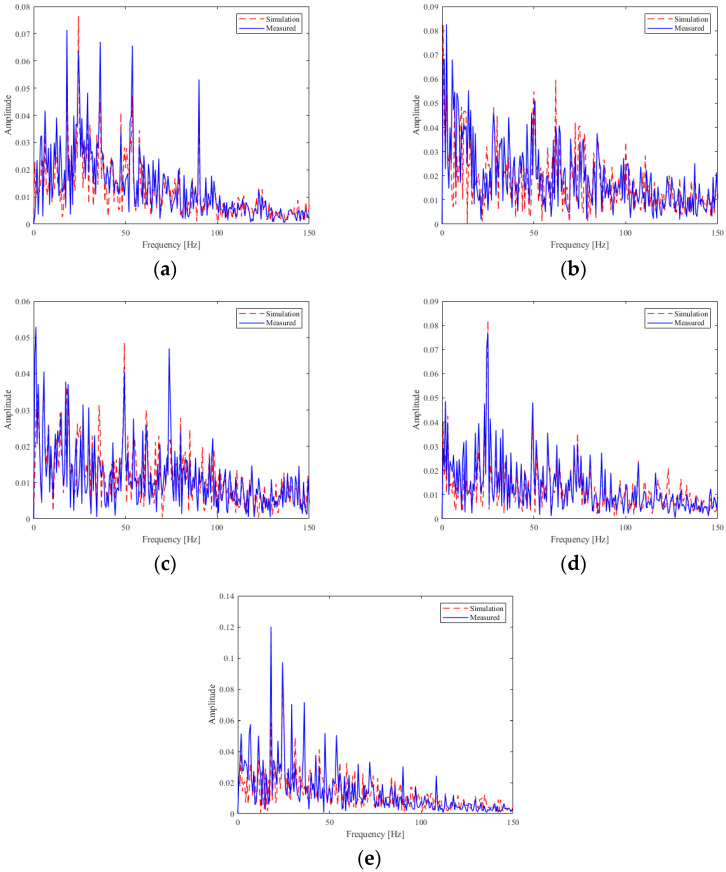
Frequency domain comparison of five gear faults: (**a**) DSP, (**b**) DSP + DB, (**c**) DB, (**d**) DB + DNB, (**e**) DC.

To evaluate the consistency between the simulation and real gear fault data, we conducted a spectral comparison analysis. As shown in Figure 6, both the simulated and measured signals exhibit similar frequency-domain characteristics. This confirms that the simulation data effectively capture the key features of real gear faults, thereby validating their use in model training for domain generalization.

### 4.2. Data Preparation

#### 4.2.1. CWRU Bearing Data

The effectiveness of the proposed method is validated by the bearing datasets, which are partly derived from the publicly available dataset of CWRU. The data are acquired by accelerometers at a sampling frequency of 12 kHz.

In order to construct multi-source domains, we considered the signals obtained from various operating conditions as distinct domains. This setup allows for a comprehensive evaluation of the performance of the proposed approach across different operating conditions, thereby verifying its effectiveness. The specifics are provided in Table 4.

#### 4.2.2. Laboratory Gears Data

To further demonstrate the utility of the proposed approach, we obtained the vibration signals of six gear faults under diverse operating conditions in the experimental gear transmission system, as shown in Table 5. The six types of gear faults are normal gear, drive gear broken tooth (DB), drive gear crack (DC), drive gear tooth surface peeling (DSP), drive gear tooth surface peeling + drive gear broken tooth (DSP + DB), and drive gear broken tooth + driven gear broken tooth (DB + DNB). The gear fault vibration signals under four loads E, F, G, and H were obtained through experiment and simulation, where the E condition is the simulation signal generated by the FEM, and F, G, and H correspond to one, two, and three loads, respectively. The rotational speeds are all 1474 rpm.

### 4.3. Result Analysis

#### 4.3.1. Comparison Methods

To comprehensively validate the effectiveness of the proposed approach, several commonly employed approaches were utilized for comparison. All approaches were implemented using the PyTorch (Version 1.13.1) framework and trained on a PC with an NVIDIA GPU GeForce RTX 4060, 32GB RAM, and an Intel Core i5-12400F CPU. (1) CNN: A basic CNN model was trained in multi-source domains to directly diagnose target domain faults. (2) IRM: A classic domain generalization method, Invariant Risk Minimization (IRM) [43]. (3) DANN [36]: Classical domain adversarial neural networks learn domain-invariant features. (4) MMD: MMD trained on multi-source data. (5) JAN: Deep transfer learning with joint adaptation networks (JAN) [44] trained on multi-source data. In the comparative experiment, to ensure the fairness of the results, this study carefully designed and detailed the parameter settings for different methods. Specifically, (1) All methods were evaluated using the same dataset and metrics to maintain consistency. (2) Key parameters and optimization techniques were standardized across all methods. For example, the learning rate was set to 0.0001, the batch size to 64, using the same CNN as a classifier, and Adam was used as the optimizer. (3) To minimize the impact of random factors on the results, each method was executed 10 times, and the average performance was reported as the final result.

In these comparison methods, for highlighting the validity of simulation data, CNN, IRM, and DANN all use measured data for multi-source domain training. Both MMD and JAN use simulation and measured data training to highlight the advantages of adversarial training.

#### 4.3.2. Bearing Test Results

Using the finite element simulation model, we generated simulation signals for ten faults under working condition A. The data for the remaining three working conditions were obtained from the measured data of the CWRU. Based on the data from these four working conditions, we designed three different fault diagnosis tasks, T1, T2, and T3, as shown in Table 6. Specifically, A, B, C, and D denote various working conditions, and each working condition contains ten different types of faults.

**Figure 7:**
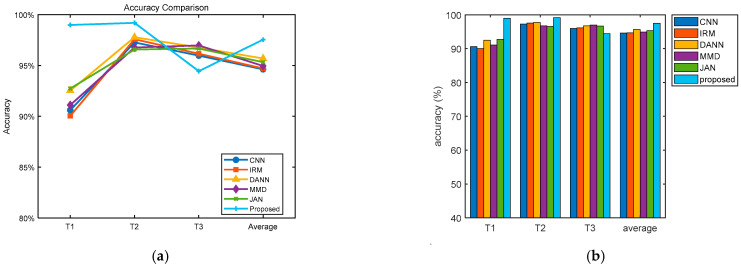
Bearing diagnostic performance comparison. (**a**) Accuracy trend changes (**b**) Overall comparison.

**Figure 8:**
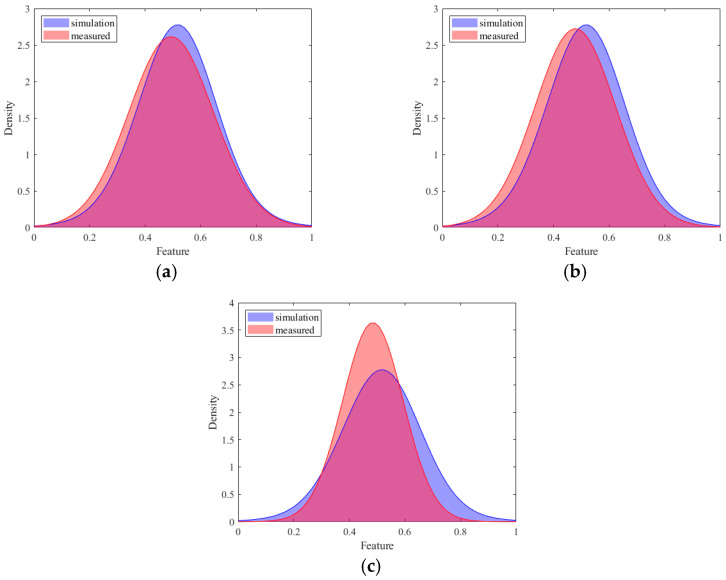
Simulation and test probability distribution of three tasks: (**a**) T1, (**b**) T2, (**c**) T3.

**Figure 9:**
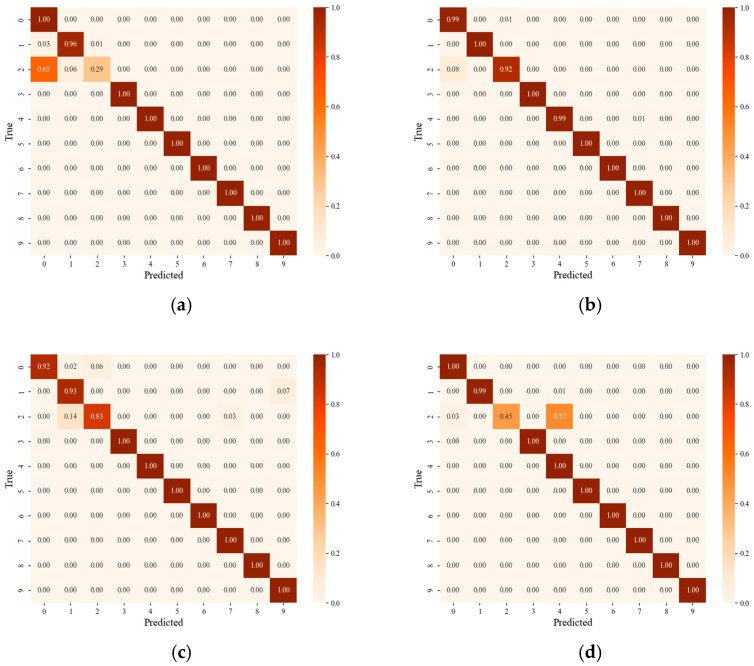
The results of four different tasks: (**a**) DANN T1, (**b**) Proposed T1, (**c**) DANN T3, (**d**) Proposed T3.

The experimental investigation is depicted in Table 7 and Figure 7. It can be observed from Figure 7 that the proposed method has the best average diagnostic performance. The average accuracy increased by 2.59% and 2.83% over the conventional CNN and IRM methods, respectively. The average accuracy increased by 1.85% compared to DANN because the source domain was enhanced with simulation data, providing diverse data which helped the model to capture common features among different domains and reinforce the commonality of features. The accuracy of the T3 task was slightly lower because the unseen test data had a large deviation from the simulated training data distribution. Taking the label 2 fault as an example, we plotted the probability distribution of the simulation data and target data of the three tasks, as shown in Figure 8. As can be seen from the figure, the distribution difference between the simulation data and the target data of the T3 task was large, which led to the accuracy of the T3 task being slightly lower than that of the first two tasks. By emphasizing the superiority of adversarial training in domain generalization and comparing it with the method based on domain adaptation, we observe that even better results have been achieved. Figure 9 shows the diagnosis results of DANN and the proposed approach in T1 and T3. As can be observed from the diagram, the main difference is in category 2. But in T3, the DANN performance was better.

#### 4.3.3. Gear Test Results

**Figure 10:**
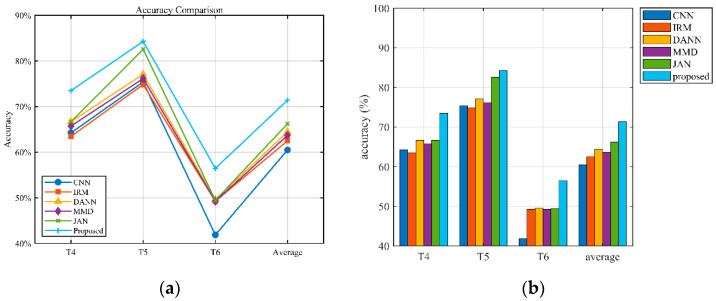
Gear Diagnostic Performance Comparison. (**a**) Accuracy trend changes (**b**) Overall comparison.

According to the gear data under four operating conditions, three different tasks were set up. As shown in Table 8, consistent with the bearing experimental setup, the simulation data are combined with the measured data to create a multi-source domain. Adversarial learning between these domains helps to learn more domain-invariant features. The experimental investigation is depicted in Figure 10 and Table 9. Relative to the experimental results of bearings, the experimental results of gears had relatively low accuracy, which was due to the large difference in the distribution of gear data under different working conditions, thus weakening the learning ability of the model to a certain extent. Relative to MMD and JAN, the average accuracy was increased by 7.7% and 5.17%, respectively.

**Figure 11:**
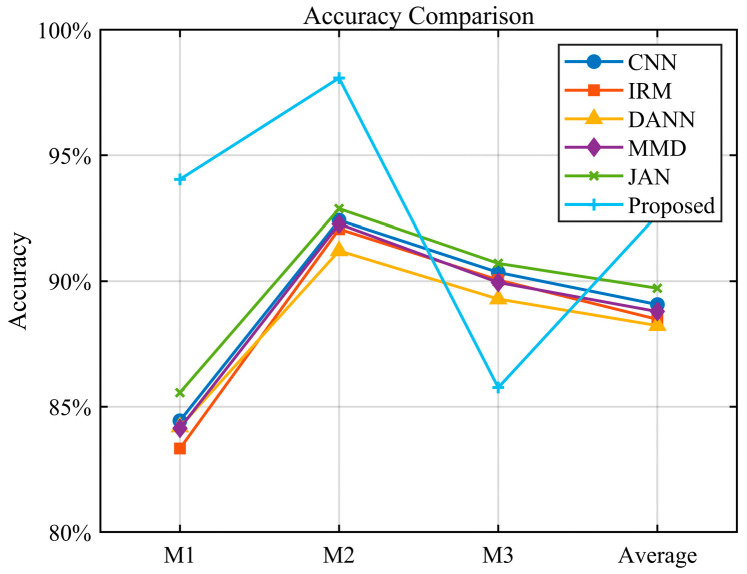
Comparison chart of experimental results.

To better demonstrate the effectiveness of the simulation data, we added simulation data to CNN and removed them from MMD. The average accuracy of the CNN increased by 2.01% in contrast to that without adding simulation data, while the average accuracy of MMD decreased by 2.9%. DANN also increased by 1.7% after adding simulation data. We designed three additional tasks to demonstrate the generalization capability of the model: M1: A, B → C, D; M2: A, C → B, D; and M3: A, D → B, C. The experimental investigation is depicted in Figure 11. Relative to the previous experiment using three conditions for training and one condition for testing, the accuracy of this experiment has decreased, but the proposed approach still shows the best results. This further shows that in the multi-source domain generalization task, making full use of rich source domain data is crucial to increasing the generalization capability of the model.

This proves the remarkable effect of adversarial training in domain generalization. Among them, all the methods had the worst effect in T6. Nevertheless, the proposed approach was superior to the comparative approach.

In summary, by effectively combining the simulation data with the actual measurement data, solid data support is provided for learning domain-invariant features in domain adversarial training. The experimental results demonstrate that leveraging simulation data to augment fault data under various working conditions can effectively tackle the issue of insufficient fault data for AI models in DG. Through domain adversarial training, the model can extract domain-invariant features for better generalization in unseen target domains. The key to multi-source domain adversarial training is the number of source domains. However, collecting fault data at various operational states under real-world conditions poses a challenge, which further highlights the importance of simulation data. Simulation data not only make up for the lack of actual data but also provide diverse training samples for the model to improve its robustness and generalization ability under different operating conditions. DG has a broad prospect in intelligent diagnosis, which is more challenging and more compatible with real-time fault diagnosis scenarios than DA. Although simulation data can help with multi-source domain data issues, different domains may have large differences in data distribution and background noise. This can make it difficult for DG models to capture common features across domains effectively.

## 5. Summary and Future Work

This study introduces a domain generalization approach for fault diagnosis utilizing simulation data augmentation to solve the problem of poor generalization performance of AI models in unseen target domains. Specifically, an FEM is used to generate simulation data of specific working conditions as an enhanced domain to promote the learning of domain-invariant features. To demonstrate the utility of this method, we conducted experimental comparisons on bearing and gear datasets. The experimental results confirm that the proposed approach can notably enhance the model’s generalization performance on out-of-distribution data. Although the effectiveness of this approach has been verified on these two datasets, there is still room for further improvement. In future work, efforts can be made to (1) reduce the discrepancy between simulation and real-world data, and (2) further validate the results using industrial field data to evaluate the stability of the proposed method in strong noise and multi-interference scenarios.

## Figures and Tables

**Table 1:** The main parameters of the network structure.

	Types of Layers	Activation Function	Kernel Size/Stride/Channel Size
	Conv1D-1	Relu	15/1/16
	BN	/	/
	Max-pooling	/	2/2/16
	Conv1D-2	Relu	3/1/32
	BN	/	/
*F*	Max-pooling	/	2/2/32
	Conv1D-3	Relu	3/1/64
	BN	/	/
	Max-pooling	/	2/2/64
	Conv1D-4	Relu	3/1/128
	BN	/	/
	Max-pooling	/	2/2/128
	Flatten	/	(128 × 4256)
*C*	FC1	/	(256,128)
	FC2	/	(128,10)
*D*	FC1	/	(256,128)
	FC2	/	(128,3)

**Table 2:** Bearing finite element model parameters.

Contact Parameters	Value	Loading Parameters	Value
Normal penalized stiffness factor FKN	0.12	Spindle gravity load	500 N
Coefficient of friction between roller and outer ring	0.016	Eccentric loads	0.12 MPa
Coefficient of friction between roller and inner ring	0.02	Speed of rotation	1797 rpm
Viscous damping ratio	0.0015	Radial preload	1 MP

**Table 3:** Geometrical and material parameters of gears.

Material Parameters	Value	Geometrical Parameters	Value
Material density	7850 kg/m^3^	Number of teeth of the master gear	55
Modulus of elasticity	2.06 × 10^11^ Pa	Number of teeth of driven gear	75
Poisson’s ratio	0.3	Module	2

**Table 4:** Description of bearing data.

Domain	Rotation Speed	Load
A	1797 rpm	0 HP
B	1772 rpm	1 HP
C	1750 rpm	2 HP
D	1730 rpm	3 HP

**Table 5:** Types of gear failures.

Failure Type	Speed	Label
Normal	1474 rpm	0
Drive gear broken tooth	1474 rpm	1
Driving gear crack	1474 rpm	2
Drive gear tooth surface peeling	1474 rpm	3
Drive gear tooth surface peeling + drive gear broken tooth	1474 rpm	4
Drive gear broken tooth + driven gear broken tooth	1474 rpm	5

**Table 6:** Fault diagnosis experiment of CWRU bearing data set.

Tasks	Source Domain	Target Domain
T1	A, C, D	B
T2	A, B, D	C
T3	A, B, C	D

**Table 7:** Diagnosis result in bearing (%).

	T1			T2			T3		
	Acc	F1	Precision	Acc	F1	Precision	Acc	F1	Precision
CNN	90.61	88.64	93.50	97.27	97.25	97.54	96.97	96.80	97.00
IRM	90.40	88.41	93.22	97.58	97.56	97.77	96.16	96.15	96.22
DANN	92.53	91.47	95.04	97.78	97.77	97.88	96.77	96.75	96.88
MMD	91.11	89.55	93.51	96.77	96.75	97.06	96.97	96.94	97.10
JAN	92.73	91.86	94.79	96.57	96.54	97.13	97.67	97.65	97.77
Proposed	98.99	98.90	99.04	99.19	99.08	99.20	94.44	93.97	96.26

**Table 8:** Gear test tasks.

Tasks	Source Domain	Target Domain
T4	E, F, G	H
T5	E, F, H	G
T6	E, H, G	F

**Table 9:** Diagnosis result in gear (%).

	T4			T5			T6		
	Acc	F1	Precision	Acc	F1	Precision	Acc	F1	Precision
CNN	64.23	58.15	56.68	75.38	73.54	80.05	41.86	31.20	26.83
IRM	63.45	57.88	72.42	74.81	73.44	79.09	49.24	30.66	33.46
DANN	66.67	60.08	79.39	77.08	73.25	84.53	49.62	35.96	34.37
MMD	65.72	61.18	72.11	76.14	75.74	77.41	49.24	38.22	33.26
JAN	66.67	59.00	70.85	82.58	77.47	90.67	49.43	37.29	32.96
Proposed	73.48	69.07	82.61	84.28	83.80	87.02	56.44	47.96	64.28

## Data Availability

Data will be made available on request.
